# Structural Changes and Operational Deterioration of the Uf Polyethersulfone (Pes) Membrane Due to Chemical Cleaning

**DOI:** 10.1038/s41598-018-36697-2

**Published:** 2019-01-23

**Authors:** Beata Malczewska, Andrzej Żak

**Affiliations:** 1 Institute of Environmental Engineering, Faculty of Environmental Engineering and Geodesy, Wroclaw University of Environmental and Life Sciences, pl. Grunwaldzki 24, 50363 Wrocław, Poland; 2 Department of Material Science, Welding and Strength of Material, Faculty of Mechanical Engineering, Wroclaw University of Science and Technology, Smoluchowskiego 25, 50370 Wrocław, Poland

## Abstract

Microfiltration (MF) and ultrafiltration (UF) membranes are capable of rejecting most of particulate and colloidal matter from natural water. The major impediment to their applications is fouling caused by contaminants that accumulate on and/or inside the membrane. Therefore, most membranes are subjected to chemical cleaning procedures as one of the methods to control fouling. Exposure to chemical cleaning agents can reduce the performance and lead to the degradation of polyethersulfone (PES) ultrafiltration membranes. This study was conducted in order to evaluate the effect of cleaning agents on the properties of the PES membranes during exposure to short-term foulant and a cleaning agent. The cleaning agents evaluated were NaOH, HCl and NaOCl. The accelerated static cleaning conditions showed significant changes in PES flat sheet membrane properties in case of cleaning with NaOCl. These changes were analyzed using SEM microscopy, FTIR spectroscopy, contact angle measurement and hydraulic membrane performance evaluation.

## Introduction

Ultrafiltration (UF) membranes are widely used for water treatment because of their ability to effectively remove significant amount of contaminants while maintaining high throughput of water at the same time. Moreover, the UF systems are often used as a pre-treatment upstream unit prior to reverse osmosis (RO). The obstacle limiting the performance and even wider adoption of UF membranes is their tendency to become fouled, resulting in a loss of water production capacity and an increase in the frequency of hydraulic and chemical cleaning^1–3^. Fouling leads to a significant increase in hydraulic resistance, manifested as permeate flux decline or transmembrane pressure increase, leading in turn to an increase in operational and material costs. Although membrane fouling can be reduced by chemical cleaning, when applied repeatedly, it exacerbates membrane ageing.

The degree of fouling is a complex function of feed characteristics, membrane properties and operating conditions^4–6^. Fouling of UF membranes is classified in a variety of ways. For example, reversible or irreversible^7,8^. Reversible fouling is defined as a fouling that can be removed by a strong shear force or backwashing. In contrast, irreversible fouling is caused by a strong attachment of particles, and cannot be eliminated by physical cleaning^9^. In addition depending on the nature of foulants, researchers often distinguish among the colloidal fouling, biofouling, organic fouling and inorganic fouling (i.e. scaling). Membrane fouling is typically controlled either by preventing it from occurring by operating the system below the defined flux, adding chemicals (especially to prevent inorganic scaling and fouling) or applying pretreatment, or by cleaning.

One of the strategies to reverse fouling employs repetitive cycles of cleaning^10^. There are four basic methods of cleaning: physical, chemical, physico-chemical and biological. When physical cleaning alone is not sufficiently effective, it is common to clean the membranes chemically^3,11–13^. The choice of a chemical cleaning agent depends on the type of fouling mechanism and the type of membrane. Concentration, cleaning time, temperature, and hydrodynamic conditions during the cleaning are further important factors affecting the efficiency of the process. Optimization of the cleaning protocols requires a deep understanding of complex interactions between the foulant and the membrane^3,7,10,14–21^.

Chemical cleaning usually removes the foulants and restores the primary performance of the membrane. At the same time, repetitive cycles of chemical cleaning could result in degradation of the membrane and, over time, the decrease of its performance. Understanding the cause of that decrease is essential for extending the lifespan of the membrane.

Polyethersulfone (PES) membranes are widely utilized for UF water treatment as well as pretreatment before RO, because they have a high hydrolytic strength and thermal resistance over a wide range of pH (from 2 to 12). Typically, PES membranes are also relatively hydrophobic^16,18,22^. Regardless of PES properties, that enable them withstand strong cleaning chemicals, a longer or cyclic exposure to chemicals may still have an impact on its lifespan.

The first step of the membrane cleaning is choosing the appropriate cleaning agents, which often entails a trial-and-error approach. Several reports describe the cleaning procedures using acid, alkali, high temperature, enzymes, and hypochlorite, and many membrane suppliers recommend sodium hypochlorite (NaOCl) as the best cleaning agent. However, a few reports have suggested that exposure to NaOCl causes changes in the membrane integrity^15–19,23^.

This study was conducted in order to evaluate the effect of cleaning agents on the properties of the PES membranes during exposure to short-term foulant and a cleaning agent. Several chemicals routinely utilized to clean membranes, including hydrochloric acid (HCl), sodium hydroxide (NaOH) and sodium hypochloride (NaOCl) were studied. The foulants were identified and the influence of the cleaning agent on changes in PES membrane morphology and loss of membrane integrity were evaluated. A key difference between this and earlier studies is the application of moderately mild cleaning conditions and the evaluation of the membrane degradation at the stages of relatively low rate of normalized permeability loss.

## Material and Methods

### Membrane

Flat-sheet polyethersulfone (PES) UF membranes (Mirodyn-Nadir GmbH, Germany) with a molecular weight cut-off of 150 kD were used for this study. The analyzed PES membrane consists of a thin and dense surface skin on top of a porous substructure residing on a non-woven web reinforcing the entire fabric. The membrane unit was operated in a dead-end mode using 47 mm diameter discs of the membrane with the effective area of 9.62 cm^2^ fit into the filter cartridge. According to the manufacturer the allowed operating conditions span are pH of 0–14 and temperatures of 5–95 °C.

Usually, the chemical cleaning protocols applied by water treatment plants comprise the following: first, the alkaline step (usually with NaOH), followed by the acidic cleaning (typically C_6_H_8_O_7_ or HCl) and, as the last one, the disinfection^3^. Those steps can be modified depending on the feed water quality.

According to the manufacturer, optimal cleaning strategy for this membrane material should be based on the type of contamination and should be determined by pilot tests.

The cleaning strategies recommended for PES membranes are usually as follows: contact times of 20–40 min for alkaline and 15–30 min for acidic cleaners, and temperature in the range of 30–50 °C.

The objective was to identify the most effective cleaning agent and, after selecting one, to evaluate the impact of different operating conditions (e.g. soaking time or temperature) on the cleaning efficiency.

In this study, the cleaning procedure was as follows: the membranes were washed with DI water to dislodge the deposit from their surface. They were then soaked in a solution of chemicals at ambient temperature (20 ± 2 °C). The first cleaning took about 15 min, then the membrane was flushed with DI water, put back into the operating unit and then filtered again with DI water for 30 min. Further consecutive cleaning periods were extended to 30 and 60 min.

Water permeability of the membrane is one of the most commonly used evaluation methods of its operational performance. In this study, the normalized permeability during cleaning was monitored and the relative clean water flux was changed, before and after cleaning, within the limits of ±5%.

The most effective cleaning agent was selected based on the evaluation of the UV removal and the initial pressure (the return of the normalized pressure to its initial value).

### Cleaning agents

Three commercially available cleaning agents were applied: sodium hydroxide (NaOH), hydrochloric acid (HCl), and sodium hypochlorite (NaOCl). The individual cleaning agents were prepared from concentrated solutions by diluting them, so the desired concentration could be reached.

### Feed

Prefiltered (5 μm) water from the Oder River (Poland) was used as the feed. It has the average value of pH 7.68, UV_254nm_ 0.096 cm^−1^, DOC 3.17 mg∙dm^−3^, SUVA_254nm_ ~3.0 cm^−1^dm^3^∙mg^−1^. The electrical conductivity (EC) of the feed water during the experiment was between 901 and 956 μs∙cm^−1^. That parameter is related to the concentration of ions in the water and it is in the range typical of the freshwater streams.

Before starting the experiments, the membranes were soaked in deionized (DI) water for about 6 hours. Then the membranes were compacted by filtering the DI water for 30 minutes. The filtration unit operated in a dead-end mode using flat sheet PES membrane fit into the filter cartridge (Pall Co., New York, NY) at operating conditions of 100 Liters per square meter per hour (LMH) and ambient temperature (20 ± 2 °C).

### Cleaning procedure

The cleaning procedure was as follows. The membranes were washed with DI water to dislodge the deposit from their surface. They were then soaked in a solution of chemicals at ambient temperature (20 ± 2 °C). The first cleaning was taking about 15 min, then the membrane was flushed with DI water, put back into the operating unit and then filtered with DI water again for 30 min. Further consecutive cleaning periods were extended to 30 and 60 min.

The selected cleaning chemicals were examined independently to evaluate the impact of each one of them individually. Therefore, a static accelerated ageing was conducted on PES membrane for 15 minutes at ambient temperature (20 ± 5 °C) in a solution of NaOH (pH 12–13), HCl (pH 3–4) or NaOCl (pH 7–8). The initially assumed solution concentrations for evaluating the membrane samples corresponded to the levels commonly used on-site by water treatment plants: 0.2% of NaOH, 0.2% of HCl and 0.02% (200 ppm) of NaOCl. The evaluation included hydraulic changes followed by the examination of the changes of the surface (contact angle), SEM imaging and FTIR.

### Permeability

Clean water (DI) permeation tests were used to characterize the permeability of the membranes at different stages of filtration and cleaning.

Typical normalized permeability for virgin and aged membranes with respect to the Vsp (specific volume treated) is characterized by two stages. First, the relatively low rate of normalized permeability loss is observed and then its rapid decrease is recognized^23^. The presented test was conducted in the first stage of membrane degradation.

### Hydraulic performance

**Figure 1:**
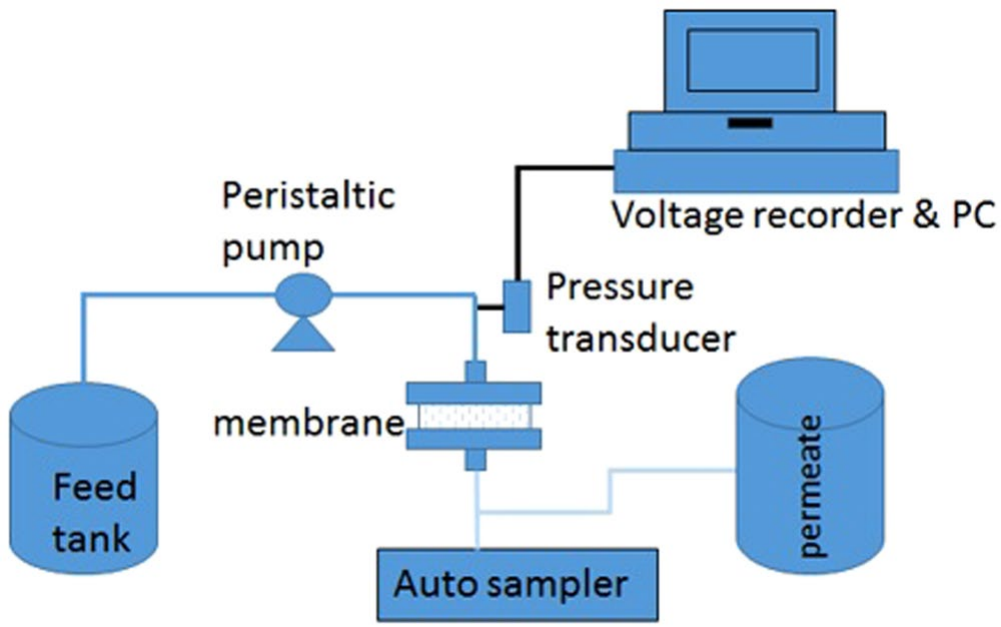
Schematic illustration of the ultrafiltration unit.

The schematic diagram of the experimental setup is shown in Fig. 1. The water was delivered to the membrane by a peristaltic pump at a constant flux (100 LMH) in a dead-end filtration mode. Permeate samples were collected for analysis. The experiments were terminated after reaching the TMP (Transmembrane pressure) limit (100 kPa).

### UV/VIS

UV absorbance spectra were recorded with Thermo Scientific™ Evolution 201 UV-Vis spectrophotometer at wavelengths from 200 to 350 nm. The UV wavelengths were selected by the presence of light-absorbing organic compounds, specifically aromatic ones. And the UV at 254 nm is a well-known parameter which is a surrogate constituent for NOM amount. The UV254 nm was monitored during the filtration in both the feed and the permeate what allows to determine percentage removal efficiency.

### Fourier Transform Infrared (FTIR) Spectroscopy

FT-IR spectra were acquired with Thermo Scientific™ Nicolet™ iZ™10 FT-IR at wavelengths from 4000 to 400 cm^−1^ with a resolution of 4 cm^−1^.

### Contact angle

The water contact angle of the membranes was measured using Drop Shape Analysis System 100 (Krüss, Germany). Each contact angle was measured 3 times (V = 10 μl) and then the average value was calculated.

### Scanning Electron Microscopy (SEM)

**Figure 2:**
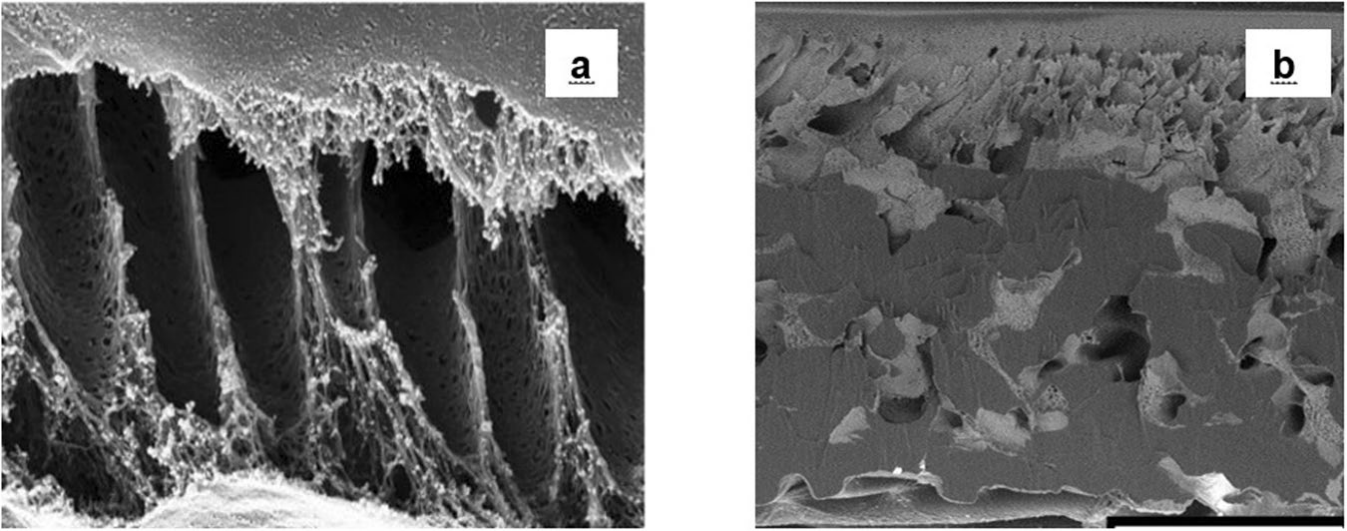
Cross-sections of PES membrane, different preparation methods; (**a**) fractured in the temperature of liquid nitrogen; (**b**) involving JEOL Cross-Section Polisher.

Many sources indicate that the SEM images of the membranes are acquired on samples cooled and fractured in the temperature of liquid nitrogen^16–20^. Such method was not satisfactorily applicable to the analyzed samples, as the fibrous material that supports the structure of membranes (a non-woven web reinforcing fabric) wasn’t brittle enough in the tested temperature. As a result, the exact details of the cross-section were indistinguishable and thus could not facilitate the proper interpretation (Fig. 2 depicts cross-section of the membrane (a) fractured in the temperature of liquid nitrogen and (b) involving JEOL Cross-Section Polisher). Therefore, a new method of preparing the samples was developed for the purpose of this research in order to properly present the intact membrane structure after preparing the cross-section.

### New methodology of preparing the samples for SEM imaging

Samples for analysis of the PES membrane morphology were prepared by cutting out a part of the membrane with a sharp razor blade. Then, the cross-sections of samples were prepared using JEOL SM-09010 Cross-Section Polisher. For each cross-section, a part of the membrane was attached to the lifting table using the mounting wax and the material at the depth of 50–75 μm was removed in order to avoid the damaging impact of the cutting blade on the edge of the sample. Finally, the polishing voltage of 4 kV was applied for the duration of 2 hours. To avoid interfering with the structure of the sample authors, decided to abandon the typical method of soaking the material in GATAN G1/G2 resin followed by wet grinding.

SEM images of both the membrane samples and the membrane surfaces were acquired by low-vacuum low-voltage Phenom G2 PRO microscope, with CeB6 filament, accelerating voltage of 5 kV and backscattered electron detector, using mixed topography-material contrast.

In order to avoid the need of additional conductive layers, which may change the morphology of the fine details, the samples were mounted in the holder by a conductive double-sided adhesive tape. That, together with the accelerating voltage of 5 kV, allowed to observe the non-conducting samples.

Additionally, the Energy-Dispersive X-ray spectroscopy (EDX) micro-analysis was applied to evaluate the elemental distributions at the microscopic scale. The EDX was performed using JEOL JED-2300 spectrometer, with 20 kV accelerating voltage, 40 μA emission current and approx. 6 μm depth of analysis. The count rate was 7000 cps and the dead time of the spectrometer was 5–10%. The qualitative analysis was performed through the comparison of the obtained spectra with KLM markers selected from a probable elements list. The analysis was performed with the ZAF method.

For the EDX analysis the samples were covered with a copper conductive path and then coated with a semi-conductive coal layer. The thickness of the layer was 30 nm and it was exposed to the vacuum of 1,3 ● 10^−4^ Pa (10^−6^ Torr) utilizing the thermal evaporation method.

The innovative approach developed in this study used a different method to get a clear cross-section of the membrane. JEOL Cross-Section Polisher enables reducing the impact of the preparation process on the microstructure of the sample. It was possible to achieve a clear cut through the sample instead of creating a large fractured area, which is usually the result of cooling the sample to the liquid nitrogen temperature followed by fracturing. Also, acquiring the images of the sample immediately after its trimming yields the microscale view of the surface directly impacted in the crumple zone. That could easily lead to misleading conclusions as to the effect of the chemical treatment on the membrane structure.

## Results

When water was filtered through the pristine membrane, small quantities of NOM were removed from the feed (based on UV_254nm_ absorbance). Typically, 25% of the UV_254nm_ was removed by the pristine membrane. Such effectiveness increased when the membrane was treated with HCl (34%), NaOH (38%), or NaOCl (40%).

**Figure 3:**
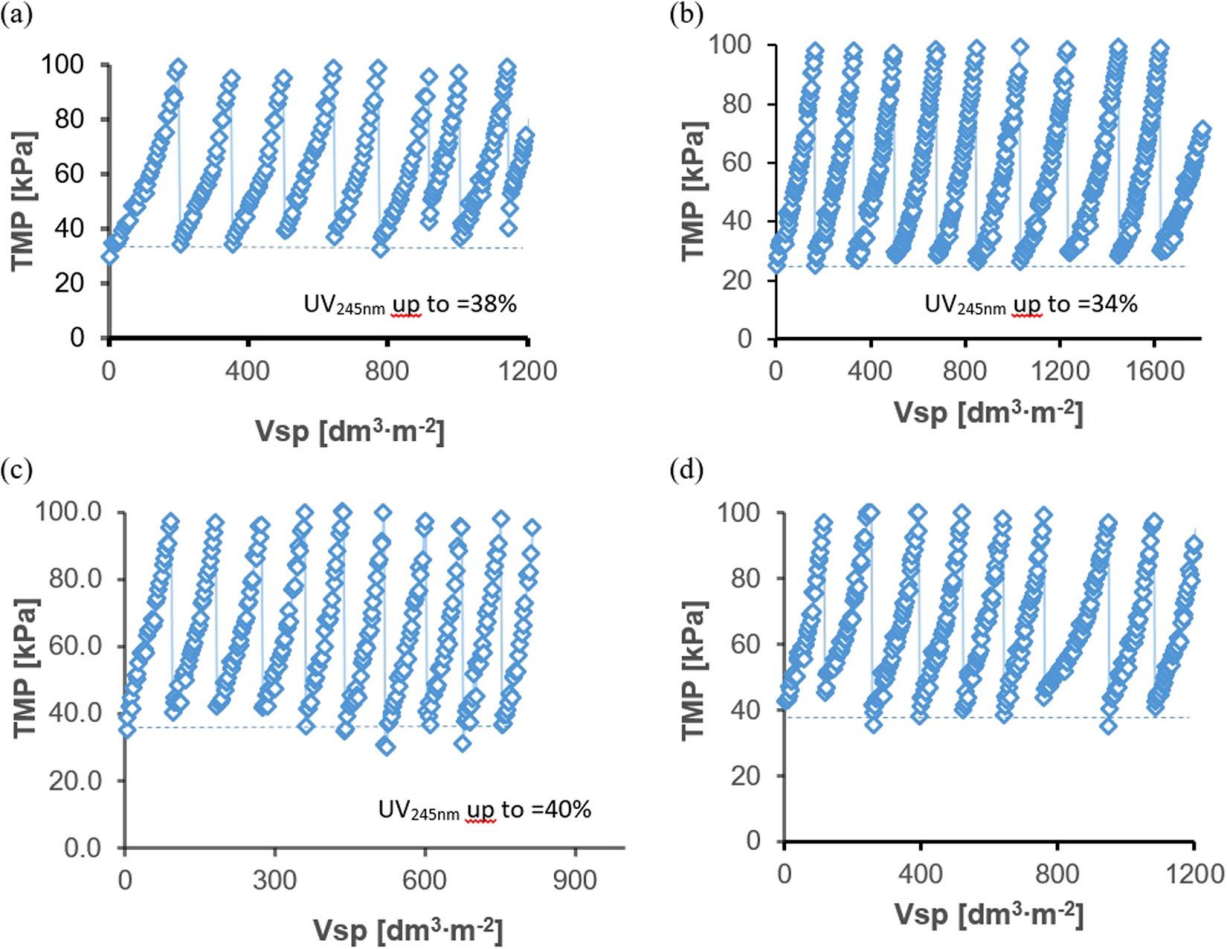
Example of TMP changes in various test systems as a function of specific volume treated (Vsp) defined as the cumulative volume filtered per unit area of membrane; (**a**) PES membrane cleaned with 0.2% NaOH, (**b**) PES membrane cleaned with 0.2% HCl, (**c**) PES membrane cleaned with 200 ppm of NaOCl, (**d**) PES membrane cleaned with 100 ppm of NaOCl.

After cleaning with NaOH or HCl, the initial TMP in subsequent consecutive cycles was the same as at the beginning of the experiment (Fig. 3), but the filtration cycles were shorter because the TMP limit was reached sooner. Similar filtration pattern was observed during NaOCl treatment of 0.02% (200 ppm) and 0.05% (400 ppm). In the case of treatment with 0.01% of Cl^−^ the cleaning procedure failed to bring the pressure down to the starting point, and a fouling pattern was observed. It is assumed the retained contaminants fouled the membrane and caused an increase of filtration resistance over time.

### FTIR

**Figure 4:**
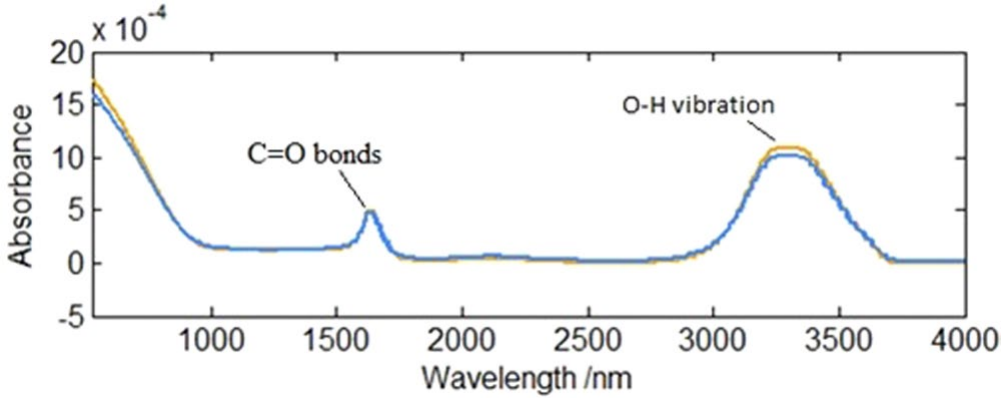
FTIR spectra of the feed and permeate, normalized by the area.

The identification of functional groups for the PES membrane was verified by FTIR spectroscopy. Samples of both feed and permeate showed a very broad band in IR range of 3000–3500 cm^−1^, and the second clear band appeared at 1642–1670 cm^−1^. In both cases, the peak in the 3300–3400 cm^−1^ range is typically associated with the O-H vibrations in water and the carbohydrate-like organic matter. The second peak corresponds to the strong hydrogen bending mode. It can also be assigned to stretching vibration of C=O bonds connected to primary amides of proteins. Figure 4 shows the spectra of feed (orange line) and permeate (blue line). The FTIR signals were normalized by assigning the highest peak which in all cases had a broad band of 3000–3500 cm^−1^ which is typically associated with the O-H vibrations in water and carbohydrate-like organic matter^24^.

**Figure 5:**
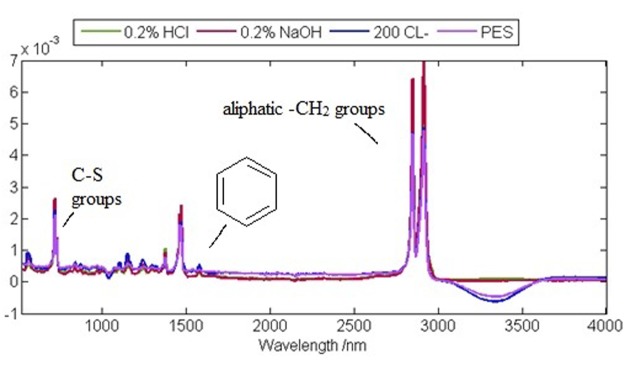
Representative FTIR analysis of the pristine PES membrane and the membrane after.

The spectra of the individual membrane samples, each subjected to a different chemical treatment, are presented in Fig. 5. The pristine PES membrane structure consists of a benzene ring, a sulfone, and an ether bond^25,26^. Usually the peaks at 1107–1240 cm^−1^ are ascribed as the bands of aromatic ethers and sulfonyl groups of PES. The band at 717 cm^−1^ is due to the C-S groups, while the bands at 1375 cm^−1^ and 1109 cm^−1^ are attributed to the sulfone group. The peaks at 1462 cm^−1^ and at 1472 cm^−1^ are indicative of alkanes.

The changes in the peak heights were small when the membrane was treated with HCl and NaOH. The increased absorbances at 2800–2900 cm^−1^ (Fig. 5 - the red curve), that are likely to be associated with the vibrations of aliphatic -CH_2_ groups, are clearly visible. However, the most significant changes in the intensities were observed when the membrane was treated with NaOCl. Specifically, the peak at the wavelength of 1042 cm^−1^ had decreased (Fig. 5), indicating the alteration of the PES sulfonic groups.

The degradation of a PES membrane evaluated by FTIR spectroscopy indicated a possible chain scission of ether sulfone, with the formation of phenyl sulfonate. The mechanism of the chain scission is usually explained by deprotonation of -CH_2_ when an alkali attacks the membrane chain structure and the formation of C=C double bonds, due to the dehydrofluorination process^3,15^.

### Contact angle

The nature of the membrane surface was investigated by the contact angle measurement. The contact angle is defined as the angle formed by the intersection of the liquid-solid interface^27^. Changes in the solid surface tension, especially the decrease in the surface energy of solids result in alteration of the contact angle. Yuan and Lee (2018) related those changes with increasing hydrocarbon layer thickness^28^. According to Lee *et al*. (2004) the contact angle of more than 60° is representative of a hydrophobic membrane^29^. In this study, the top surface of the pristine membrane had the contact angle of 50.9 ± 2.4° and the bottom surface of 68.5 ± 0.9°, so the dense surface skin of the membrane is likely hydrophilic.

Fouling significantly increased the contact angle of the PES membrane. After filtration of the feed water it had increased to 71.1 ± 4.1°. The cleaning agents were found to modify the membrane surface as well. The biggest increase (up to 36% on the top, and 48% on the bottom) of the contact angle was recorded for the fouled membrane treated with NaOCl. As for NaOH, the increase of the contact angle, on the top surface, was in the range from 61.3 ± 2.3° to 62.1 ± 2.7°. The smallest increase in the contact angle was observed after the membrane was treated with HCl, and at concentration of 0.2% it was 53.6 ± 2.1°.

### SEM

**Figure 6:**
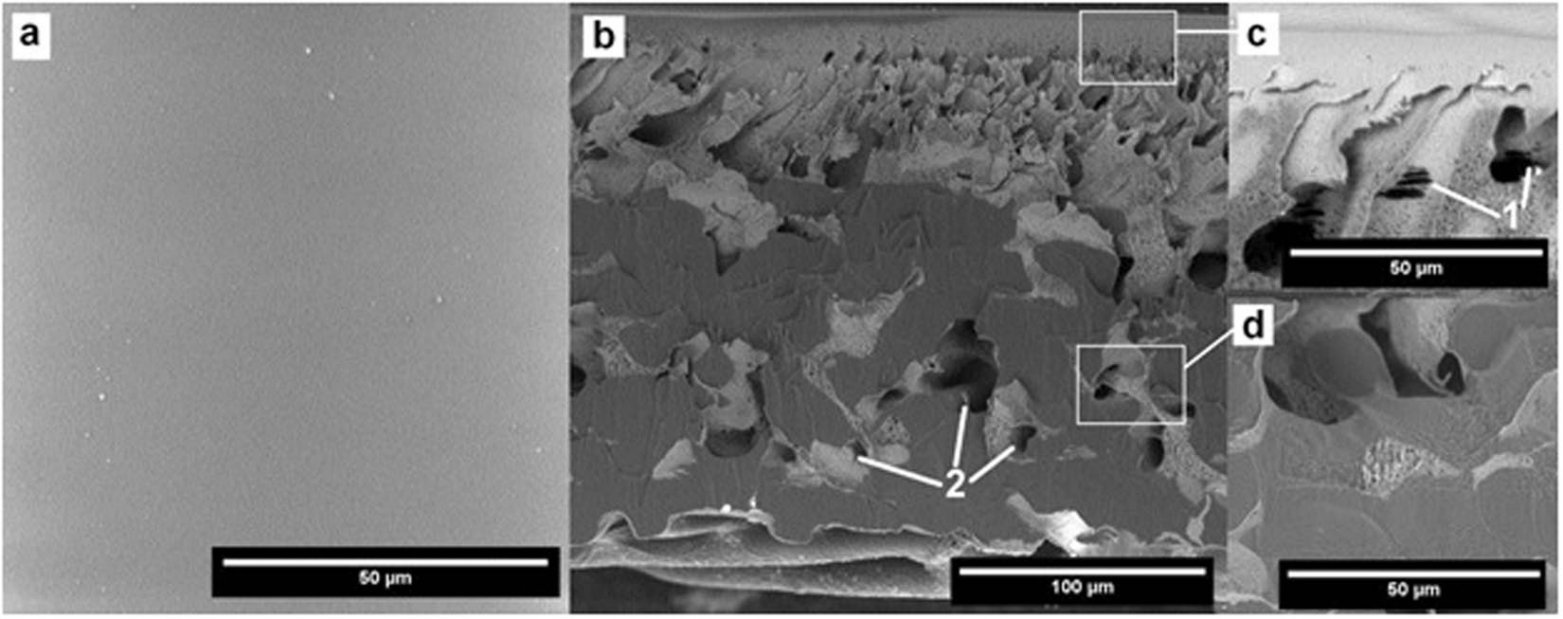
SEM images of (**a**) the pristine membrane’s surface, (**b**) cross-section, (**c**) magnified (2X) surface skin, (**d**) magnified (2X) porous substructure.

**Figure 7:**
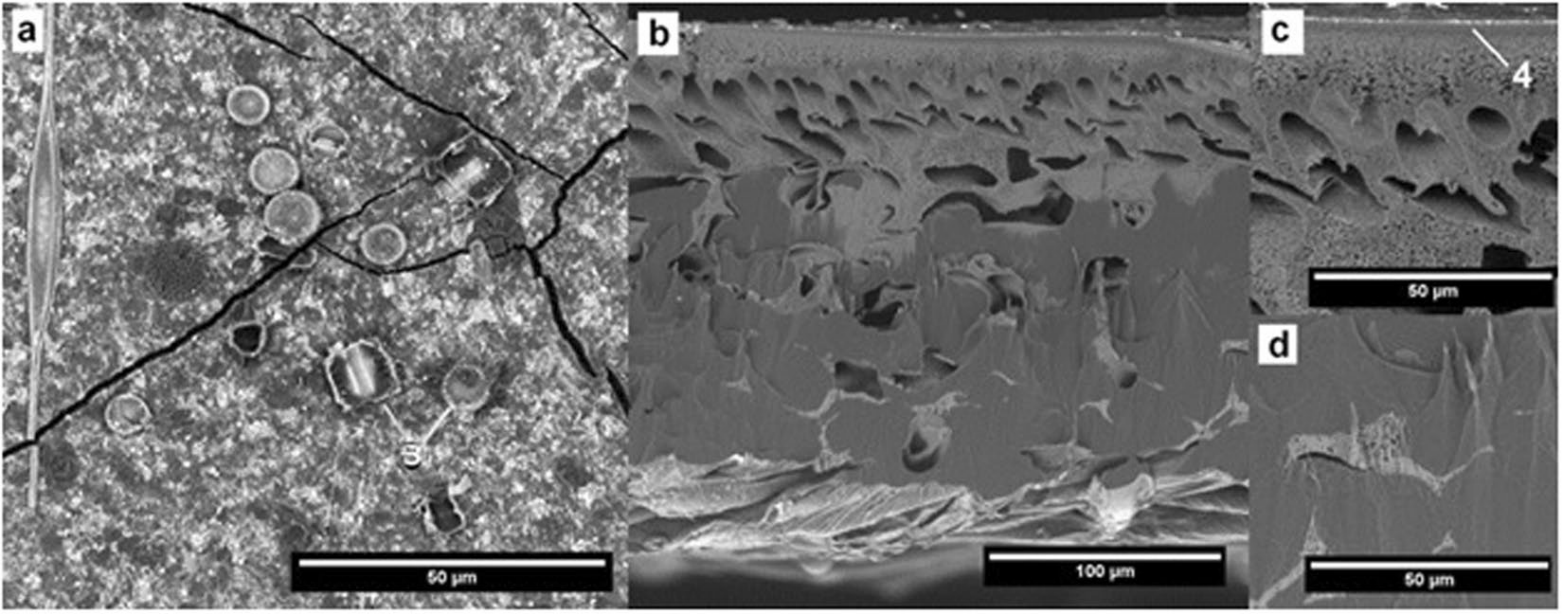
SEM images of the fouled membrane.

**Figure 8:**
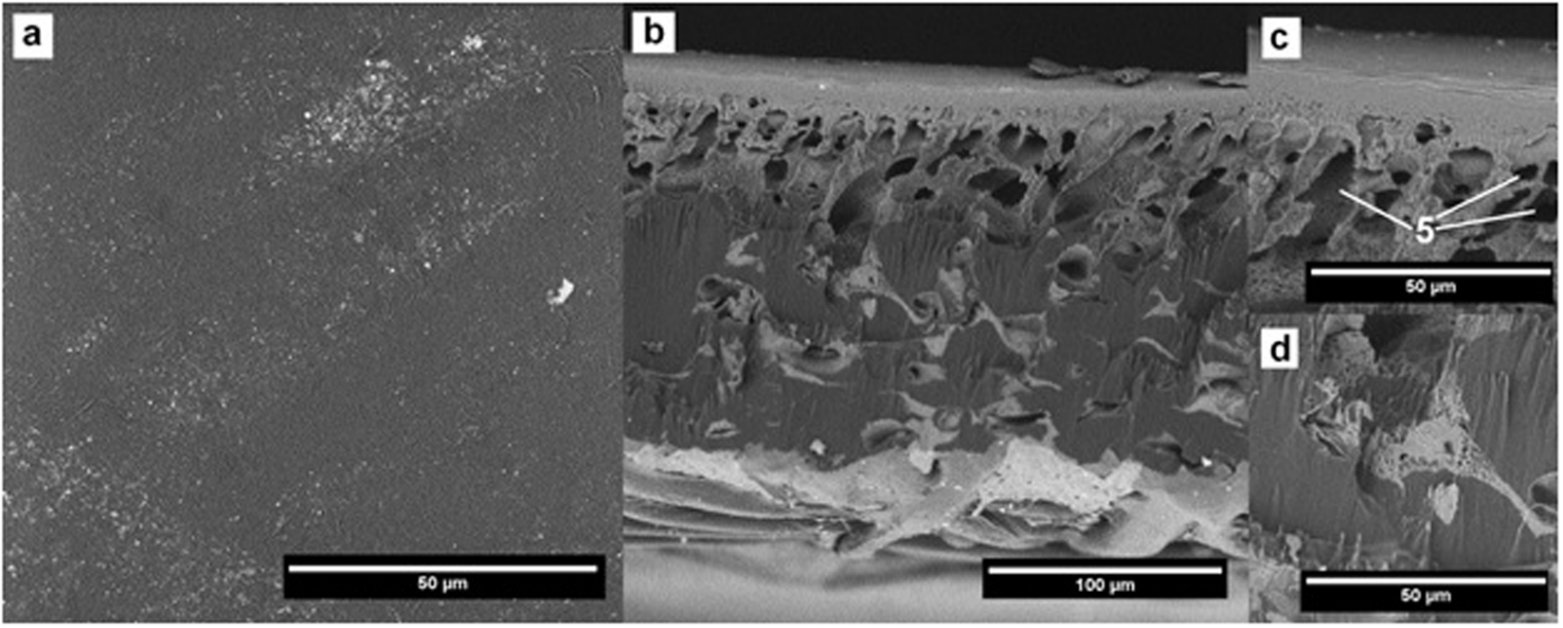
SEM images of the membrane after NaOH cleaning.

**Figure 9:**
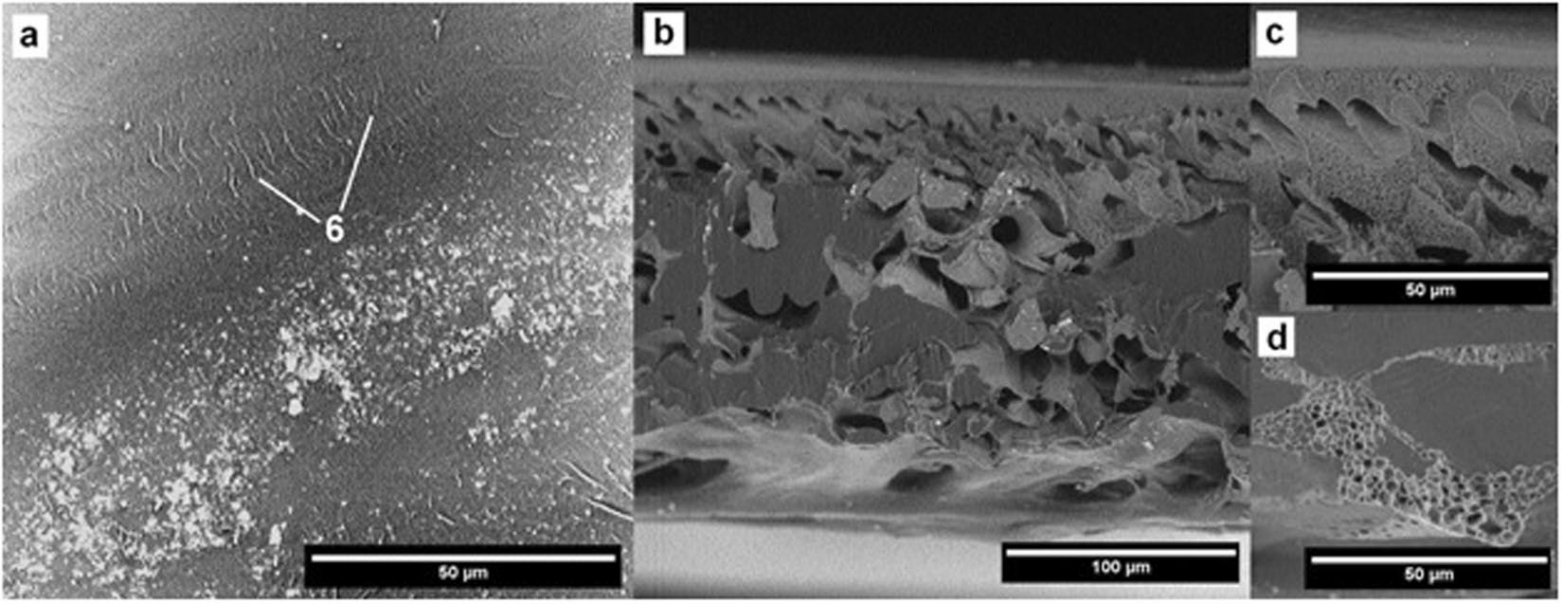
SEM images of the membrane after HCl cleaning.

**Figure 10:**
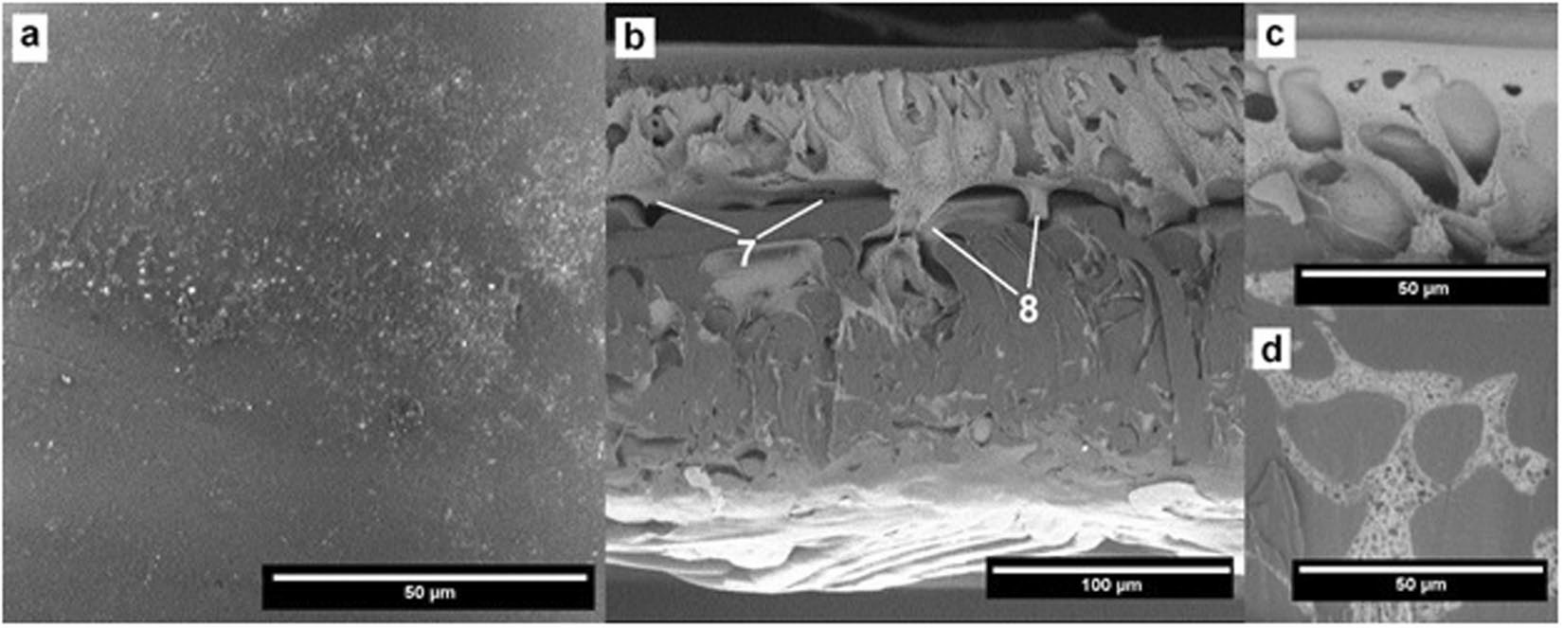
SEM images of the membrane after NaOCl cleaning.

**Figure 11:**
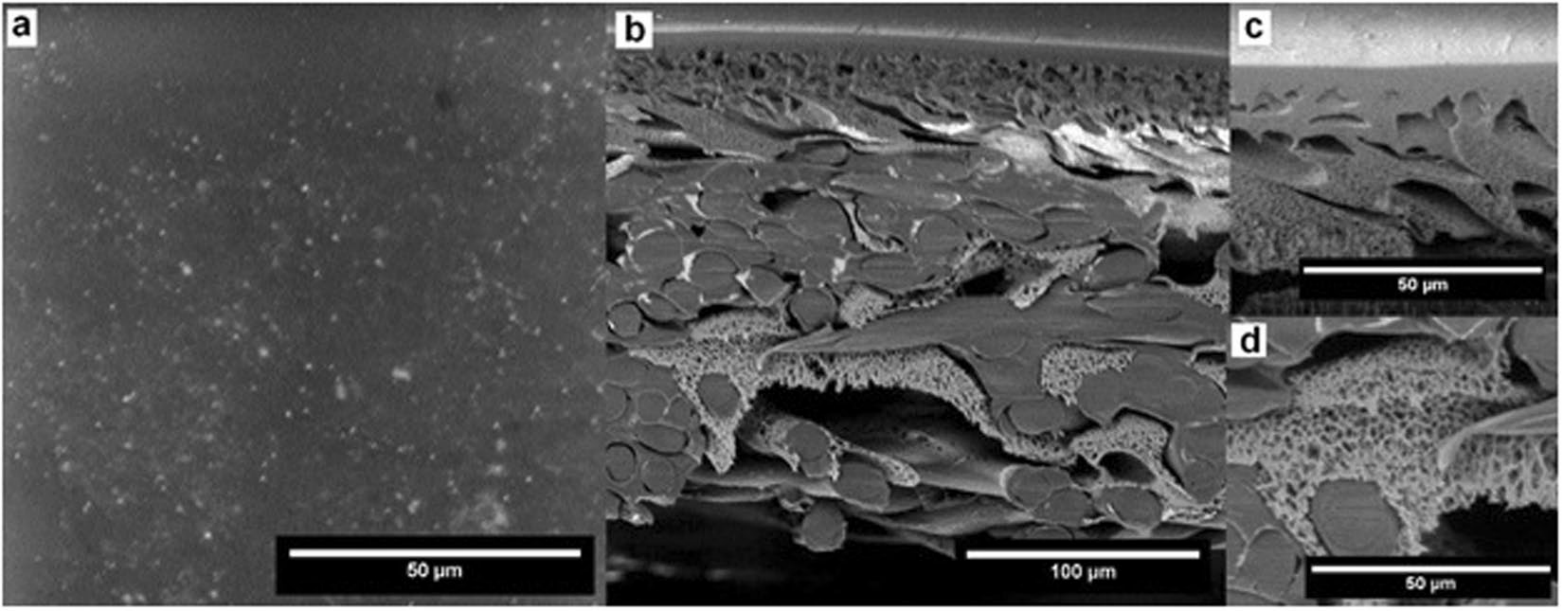
SEM images of the membrane after treatment for 15 min with 100 ppm of Cl^−^ NaOCl.

**Figure 12:**
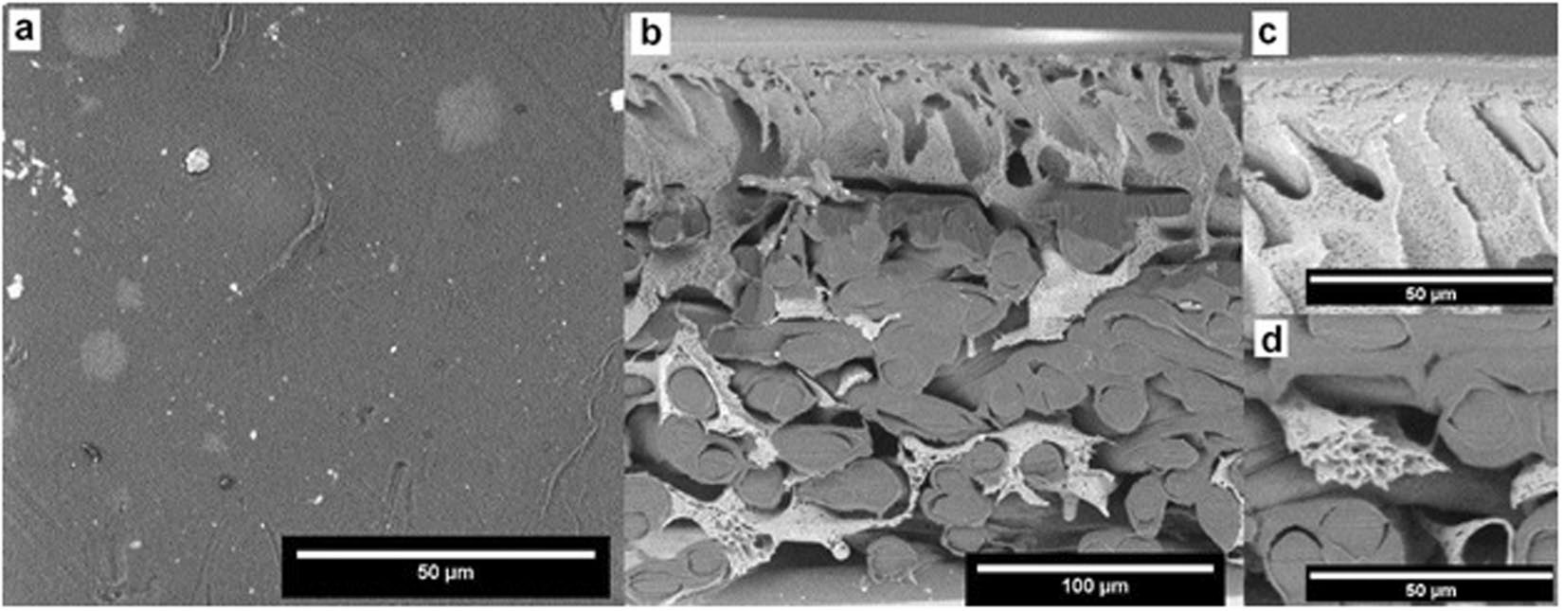
SEM images of the membrane after treatment for 15 min with 400 ppm of Cl- NaOCl.

Interactions between the cleaning agents and the membrane morphology were analyzed by SEM (Figs 2,6 to 12). Figures 6 to 12 present the SEM images of the surface (a) and the cross-section (b) of the PES membrane investigated in this study. The enlarged images present the magnified view of the dense surface skin (c) and the porous substructure (d). The approximate locations of the (c) and (d) images are indicated in Fig. 6b and are shown for each membrane.

### The pristine membrane

The pristine membrane before exploitation is characterized by a clean outer surface (Fig. 6a), and a certain amount of microscopic porosity within the cross-section of the sample (Fig. 6b). Below the dense surface skin the porous filtering material with pillar structure can be observed as well as, in the layer located directly beneath the surface skin, small gaps of the filtering material (Fig. 6c, pointed by 1). They can probably be attributed to the folding of the UF membrane sheet in transportation.

The upper filtration layer with a thickness of about 70 µm is supported with the non-woven web reinforcing fabric. The connection between both layers can be characterized by a high relative compactness, and by the presence of junctions of the porous substructure penetrating the supporting fabric.

Below the porous substructure, at the bottom of the UF membrane, are open voids visible between the fibers (Fig. 6b, pointed by 2).

### Fouled membrane

The outer surface of the fouled membrane shows the surface evenly covered with adsorbed contaminants and some planktonic diatom species (e.g. *Diatoma vulgaris* and *Melosira varians*) (Fig. 7a, pointed by 3). The contaminants are fractured by a web of cracks likely formed when drying the sample. The cross-section of the sample, slightly magnified, reveals no microscopic differences when compared to the pristine membrane. The magnified section of the outer layer of the surface skin shows the evidence of contaminants layer with a thickness of about 3 µm (Fig. 7c, pointed by 4). Further below, a blockage of a significant part of the pores in the filtration layer has occurred. The blockage is also noticeable in the porous substructure. The lower part of the membrane does not reveal any significant contamination.

### Fouled Membrane treated with NaOH

The outer surface of the membrane treated with NaOH shows a small amount of adsorbed impurities on the surface and there are no visible signs of degradation of the top layer (Fig. 8). Pillar-like structure of the top filtration layer reveals an increase in the size of cavities while the micro-pores appear intact (Fig. 8c, pointed by 5). The interface between the reinforcing fabric and the porous substructure does not show a significant difference in its construction when compared to the pristine membrane.

### Fouled membrane treated with HCl

The surface of the sample treated with HCl demonstrates the greatest amount of adsorbed pollutants on the surface. It is also possible to observe creases on the dense skin surface (Fig. 9a, pointed by 6).

The degradation of porosity in the filtration material is visible both at the top and at the bottom of the membrane. It indicates a significant alteration of the membrane structure.

### Fouled membrane treated with NaOCl

The surface treated with NaOCl shows a small amount of adsorbed impurities on the membrane surface (Fig. 10a). In the top layer, the degradation of the structure’s porosity can be observed along with a slight increase in the size of cavities present in the reinforcing fabric of the filtering material. More importantly, a significant delamination of the top layer of the filtering material from the supporting fabric is clearly visible (Fig. 10b, pointed by 7). The connection between both layers is held only by thin “bridges” of the filtering material (Fig. 10b, pointed by 8). This significantly deteriorates the mechanical properties of the membrane and poses a threat of a complete delamination of the top layer from the support structure.

The efficiency of chemical cleaning depends on many factors, including chemical concentration, pH, cleaning temperature, and cleaning time^17,18^. Authors have shown that the exposure to sodium hypochlorite is the most effective in foulants removal but, at the same time, it also severely impacts the membrane’s morphology. This finding is consistent with the previous works^15,17–19^ reporting the impact of NaOCl on the membrane performance.

In the presented research, in spite of moderately mild cleaning conditions, the membrane is still affected and the water permeability increases about 5% after soaking in sodium hypochlorite.

The contact angle and the FTIR spectra indicated that the alteration of the chemical composition of the membrane causes a small reduction in the degree of hydrophilicity. SEM evaluation of the tested membranes indicated a small deterioration of the membrane structures, especially after NaOCl was used.

Of all the cleaners studied for the PE UF membrane, sodium hypochlorite 0.02% available chlorine) was the most effective, and, at the same time, it induced the degradation of the studied membrane the fastest.

### The effect of concentration

Relative DI water permeability for a static immersion in NaOCl solution of 0.01%, 0.02% and 0.04% at pH between 7 and 8 was first assessed for the period of 15 min. The overall average removal of UV_254_ had improved by 20% when the soaking solution concentration was increased. As it was expected, the NOM adsorption increased with a higher dose of NaOCl, since NaOCl removes membrane foulants by oxidation reactions, and the foulants become water-soluble so they can be easily detached from the membrane^7,30^. Additionally, it confirms the hydrophilization of the membrane surface reported by^31^.

Representative samples of changes in the morphology and the structure of the membrane treated with 0.01% as Cl^−^ and 0.04% as Cl^−^ are presented in Figs 11,12).

**Figure 13:**
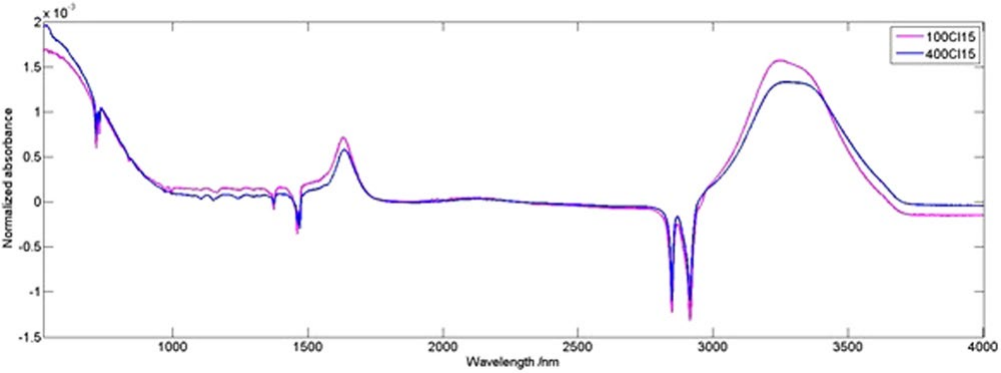
FTIR analysis of the membranes treated with 100 ppm and 400 ppm NaOCl as Cl^−^ (15 min), normalized by area.

There were visible changes in the peak heights and numbers when the membranes were treated with different concentrations of NaOCl solutions. The FTIR spectra presented in Fig. 13 show the changes in the region of 1400 and 1770 cm^−1^ for the PES membrane treated with 0.04% and 0.01% (of Cl^−^).

The contact angle of the membrane treated with 0.01% as Cl^−^ was 65.6 ± 3.4° on the top surface and 77.8 ± 2.3° on the bottom surface. In the case of 0.02% as Cl^−^ it was 69.3 ± 2.4° and 101.8 ± 1.2° on the top and the bottom surface, respectively. Finally, for the concentration of 0.04% as Cl^−^ it was 73.3 ± 1.0° and 106.6 ± 2.4° on the top and the bottom surface, respectively (Table 1). The contact angle increased with higher NaOCl concentrations, when the time of 15 min was applied.

### The effect of time

The soaking time is another important factor in efficient cleaning. Typically, the longer the time of the cleaning the higher its efficiency^15^ and, as it was expected, the overall average removal of UV_254_ was better when the soaking time was prolonged, by 42% for 30 min and by 45% for 60 min (Table 1).

As shown in Table 1, treatment with a more concentrated NaOCl solution in a prolonged time of exposure tends to increase the contact angle of the membrane, especially on the top surface.

**Table 1:** Changes of the contact angle of the membranes treated with different concentrations and different times.

Time:	15 min	30 min	60 min
0.01% (100 ppm) of NaOCl			
top surface	65.6 ± 3.4°	66.9 ± 2.2°	70.5 ± 1.0°
bottom surface	77.8 ± 2.3°	103.5 ± 3.8°	103.1 ± 7.8°
0.02% (200 ppm) of NaOCl			
top surface	69.3 ± 2.4°	72.6 ± 3.4	75.7 ± 2.8°
bottom surface	101.8 ± 1.2°	94.3 ± 1.1	115.3 ± 5.2°
0.04% (400 ppm) of NaOCl			
top surface	73.3 ± 1.0°	75.3 ± 1.0°	78.8 ± 6.0°
bottom surface	106.6 ± 2.4°	96.1 ± 2.8°	115.7 ± 3.9°

**Figure 14:**
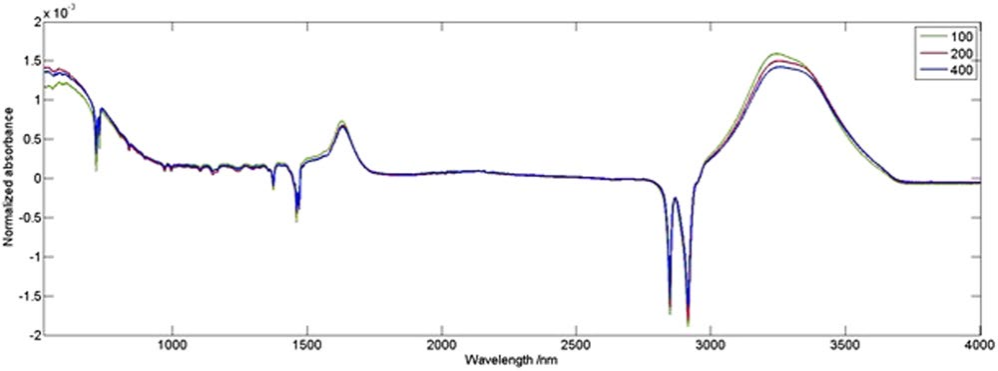
FTIR spectra of the membranes treated with 100 ppm, 200 ppm and 400 ppm of NaOCl for 15 min, normalized by area.

FTIR spectra of the very same samples are shown in Fig. 14 and similar changes in the region of 1400 and 1770 cm^−1^ were observed.

### The effect of temperature

The cleaning solution’s temperature is also of importance for efficient cleaning. The solubility of salts and organic compounds is temperature-dependent and is generally found to increase with higher temperatures^3,14^. To explore the impact of an increased temperature on cleaning efficiency of the PES membrane the samples were soaked for the duration of 15 min in the cleaning solution at the temperature of 50 °C.

The UV_254_ profiles fluctuated at the beginning of the test, but then they stabilized and the overall average removal over the whole experiment was improved by 20% when the temperature was elevated to 50 °C. The most significant effect of increasing the temperature of the cleaning solution was recorded when 0.01% of NaOCl was applied.

The contact angle value exhibited a variations of the data as well. For the membrane treated with 0.01% as Cl^−^ it was 61.2 ± 6.8° on the top surface and 83.5 ± 3.7° on the bottom surface. In the case of 0.02% as Cl^−^ it was 71.6 ± 4.2° and 87.7 ± 2.4° on the top and the bottom surface, respectively. And for the concentration of 0.04% as Cl^−^ it was 58.1 ± 3.1° and 92.5 ± 7.2° on the top and the bottom surface, respectively. Generally, at higher temperatures, the contact angle’s decrease was less pronounced, which may be connected with the presence of surface impurities even after cleaning.

**Figure 15:**
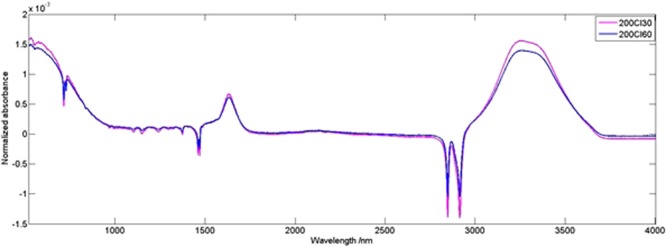
FTIR spectra of the membranes treated for 30 min and 60 min with 200 ppm NaOCl, normalized by area.

The FTIR analysis suggested that the chemical cleaning at a higher temperature did not significantly alter the chemical composition of neither the active nor the support layer of the membrane (Fig. 15).

As for the SEM analysis, it revealed the delamination of membrane layers (the outer part of the surface is slightly separated from the core). It is visible in all samples, and with the increase of time or concentration that effect is stronger. At the same time, it also increases the thickness of the entire membrane (there are increasingly more gaps between various elements of the structure).

**Figure 16:**
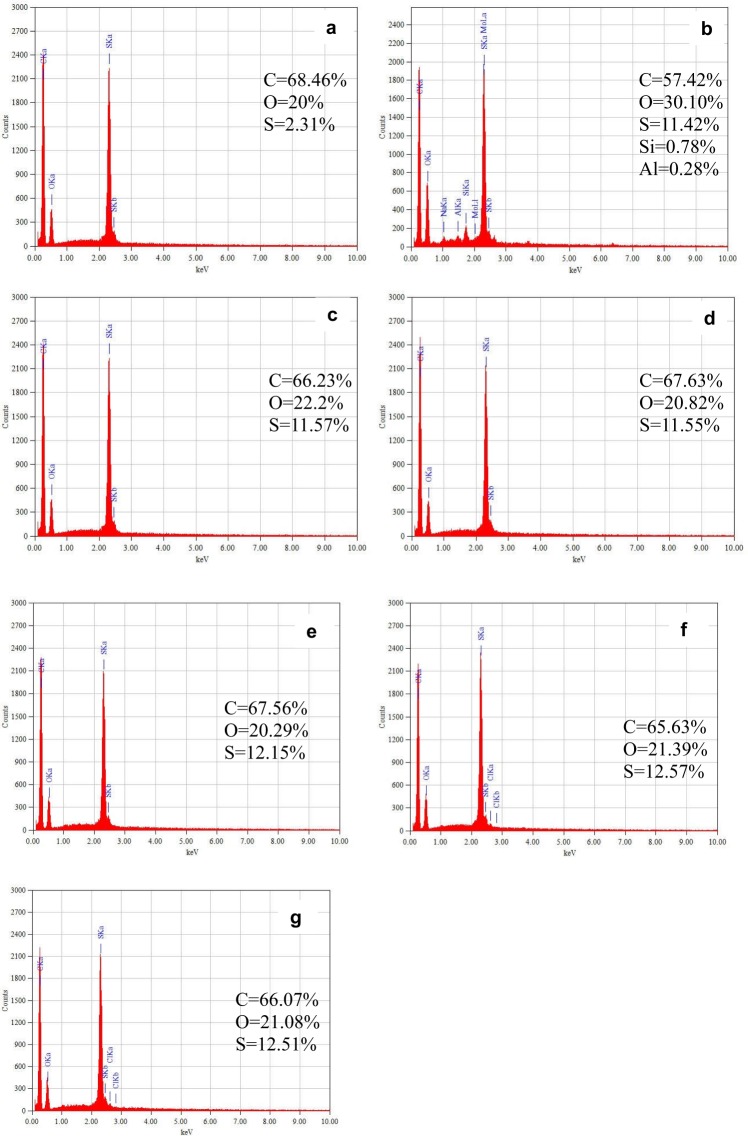
The EDX spectrum (**a**) dependence of the number of counts as a function of radiation energy) of analyzed sample: (**a**) the pristine membrane, (**b**) fouled membrane, (**c**) membrane after NaOH cleaning, (**d**) membrane after HCl cleaning, (**e**) membrane after NaOCl cleaning, (**f**) membrane after treatment for 15 min with 100 ppm of Cl^−^ NaOCl, (**g**) membrane after treatment for 15 min with 400 ppm of Cl^−^ NaOCl.

The EDX microanalysis allows for identification of chemical elements included in the tested material, but reliable quantitative analysis is possible with flat and polished samples. The EDX spectrum presented in Fig. 16 depicts the atomic composition of the evaluated membrane. The relative concentration of the elements found in the pristine PES membrane was as follows: C = 68.46%, O = 20%, S = 2.31%. Obviously, the fouled membrane was characterized by a greater diversity of elements, such as C = 57.42%, O = 30.10%, S = 11.42%, Si = 0.78%, Al = 0.28%, and Na, Ca. Unfortunately, when EDX is applied to the analysis of the light elements (like O) it also yields a loss of the measurement accuracy. Therefore, although the EDS relative errors were within the acceptable limits^32^ for all measured samples, the increase of the relative concentration of sulfur is clearly visible. Arkhangelsky *et al*. (2007) reported the elevated concentration of sulfur and carbon as an evidence of the PES chain scission during long-term exposure on NaOCl^16^.

## Discussion

At present, at lot of research is dedicated to modifications of the membrane structure or improving the pretreatment methods. The interaction between the chemicals and the structure hasn’t been of concern so far, since it has been considered one of the methods reducing the fouling. Only lately, the alterations in membranes aged in chemical solutions have become a new topic of research^3,21,23,33,34^.

Nguyen and Roddick (2011) reported that alkaline cleaning agents were more efficient than acidic agents in removing the hydraulically irreversible foulants^7^. This study confirmed that NaOH was more efficient in cleaning the PES membrane, also when applied in water treatment. On the other hand, most of the membrane suppliers recommend NaOCl as the cleaning agent. Puspitasari *et al*. (2010) revealed that NaOCl had a significant impact on the membrane structure and the impact might vary over a short or long-term exposure^17^.

Pellegrin *et al*. (2013 a,b) argued that in the case of cleaning with NaOCl, the pH of the cleaning solution is a crucial factor contributing to the polymer membrane degradation^18,19^. Also, according to Arkhangelsky *et al*. (2007) the cleaning agent’s doses have a major impact on the polymer membrane performance and properties^16^. This study can support this statement only in the case of NaOCl, as other cleaning agents evaluated did not reveal any correlation between the dosage and the membrane degradation. In the range of the analyzed concentrations it was noticed that even when mild conditions of cleaning were applied, the changes in the membrane morphology were clear. The SEM images of the surface of the sample treated with NaOCl showed the smallest amount of impurities what, combined with the increase of the contact angle, suggests changes of the membrane surface properties. Also, significant differences were observed between the cross-sections of the pristine membrane and the fouled one treated with NaOCl.

The presented research indicates that the chlorine treatment of the PES membrane impacts the membrane’s properties even when exposure to short-term on NaOCl. It becomes more hydrophilic after being exposed to chlorine. Some researchers attribute those changes to leaching of polymeric additives due to a partial scission of the C-S bond that leads to the loss of polymer integrity^15,16^.

Rouaix *et al*. (2006) reported that the exposure to sodium hypochlorite at high concentrations led to chain breaking changes in the membrane texture and its mechanical properties^15^. Furthermore, Pellegrin *et al*. (2013b) suggested, that poly(N-vinylpyrrolidone) (PVP) addition appeared to be dislodged from the PES membrane matrix and the presence of chain scission of the sulfonic acid group or the phenyl chloride group under the exposure to NaOCl resulted in the increased fouling^19^.

Zhou *et al*. (2010) proved that increasing the PES molecular weight would lead to a larger pore size in the skin layer, thereby contributing to higher permeability and lower rejection^35^. The use of NaOCl on clean, fouled PES membranes had been shown to have negative impact on the surface morphology and a long-term exposure led to the reduction of its separation performance^3,15,16,33,34^. The effect of NaOCl cleaning agent has been reported to be dependent on the working conditions, especially on the pH^22^. Studies on degradation of PES membranes by NaOCl also lead to the evaluation of degradation mechanisms.

**Figure 17:**
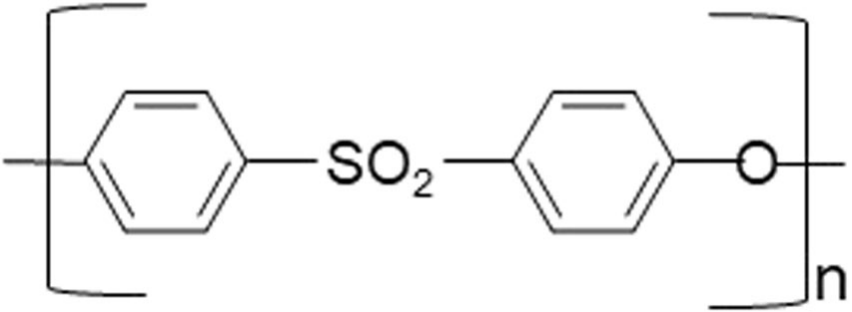
Molecular structure of PES.

**Figure 18:**
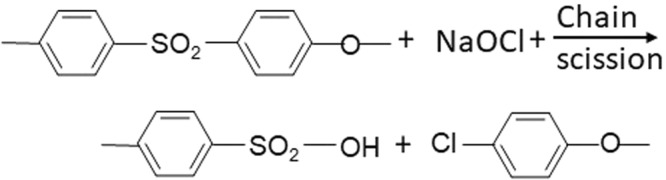
The mechanism of NaOCl attack on PES membrane according to^28^.

The schematic representation of the PES membrane’s structure is presented in Fig. 17:

In the relevant literature, there is no consensus on the mechanism of degradation by NaOCl. Two possible reactions have been proposed^28^. Gaudiche-Maurin and Thominette (2006) and Arkhangelsky *et al*. (2007) suggested that the chain scission of the PES is triggered by the partial scission of the ether-sulfone linkage^16,30^. Lately, Hanafi *et al*. (2016) and Tsehaye *et al*. (2018) evaluated the degradation mechanism, and suggested that PES chain scission and radical oxidation occurred when membrane was exposed to NaOCl (Fig. 18).

On the other hand, Yadav *et al*. (2009) suggested that each layer of the membrane should be studied individually since each layer presents different response to NaOCl exposure^34^. Additionally, the exact mechanism of the reaction for the PES membrane degradation is strongly dependent on pH and exposure time.

This study was performed at the short-term foulant and cleaning agent exposure, therefore it can’t support the theory about the chain scission. Thus it indicated there were subtle changes in the membrane morphology from the beginning of the exposure. Such changes could further lead to the irreversible degradation of the membrane performance and characteristics. In the case of NaOCl treatment, there was a clearly visible delamination of the top layer of the filtering material from the supporting fabric. Presented results confirmed the degradation of the PES membrane by NaOCl caused by the oxidation with free chlorine. In spite of restoring the pressure close to the initial value, cleaning with NaOCl also caused the consecutive runs be shorter which is indicative of the membrane degradation in the longer term.

Contact angle tests indicated that not only had the surface of the membrane been altered but also its inner structure was affected. Under the accelerated ageing conditions significant changes of the contact angle on the top and the bottom surfaces were recorded for the tested concentrations of cleaning agents.

## Conclusion

Accelerated chemical cleaning study was conducted on the PES flat sheet membranes. The properties of the pristine, fouled, and cleaned membranes were compared.

The SEM images of the pristine and fouled membranes confirmed that foulants initially settle and gradually cover mostly the membrane surface. After each of the ten cycles of cleaning a removal of most of the deposited material from the membrane surface was observed. Additionally, the FTIR analysis revealed the chemical changes in PES functional groups of the flat sheet membrane.

When comparing the results of applying each of the cleaning agents, it was confirmed that NaOCl induces the degradation of the UF PES membrane the fastest. It takes place even if mild cleaning conditions are applied.

The results showed the changes in the surface properties, morphology and hydraulic performance suggesting that NaOCl could cause ageing of the PES membrane after a prolonged exposure.
